# Risk of carpal tunnel syndrome among patients with osteoarthritis: a US population-based study

**DOI:** 10.1186/s12891-024-07459-1

**Published:** 2024-06-15

**Authors:** Shuang Chen, Tina Ho, Julius Asubonteng, Rachel E. Sobel, Simon Eng, Stephen J. DiMartino, Angelika Manthripragada

**Affiliations:** Regeneron Pharmaceuticals, Inc., 777 Old Saw Mill River Road, Tarrytown, NY USA

**Keywords:** Carpal tunnel syndrome, Osteoarthritis, Incidence rate, Risk factors, Real-world evidence

## Abstract

**Background:**

Carpal tunnel syndrome (CTS), an entrapment neuropathy caused by pressure of the median nerve, is a progressive condition that can lead to a decreased quality of life. Studies suggest an association between CTS and arthritis; however, previous studies examining osteoarthritis (OA) and CTS are limited in number, scope and study design. This study estimated the incidence and risk of CTS among patients with OA, both overall and by specific joints, in a large population-based cohort in the United States.

**Methods:**

Patients from the Optum claims database aged ≥ 45 years and diagnosed with OA between January 1, 2018, and December 31, 2022, were eligible for the OA cohort. The non-OA cohort included those without a diagnosis of OA at the index date and no history of OA for 12 months pre-index. Baseline characteristics were balanced using propensity score matching. The risk of CTS in the OA and non-OA cohort were evaluated using incidence rates and adjusted hazard ratios that were estimated using Cox regression.

**Results:**

After applying the inclusion/exclusion criteria, 3,610,240 of the 6,023,384 adults with a diagnosis of OA remained in the OA cohort. After propensity-score matching, each cohort included 1,033,439 individuals. The incidence rates for CTS per 1000 person-years were 7.35 (95% confidence interval [CI] 7.21–7.49) in the OA cohort and 1.44 (95% CI 1.38–1.50) in the non-OA cohort. The risk of developing CTS in patients with OA was ~ 4 times that of patients without (hazard ratio = 3.80; 95% CI 3.54–4.07). This increased risk was found across all OA joint types, with OA of the hand/wrist having the highest risk for CTS. Additionally, multiple OA joints presented a higher risk compared with a single affected joint.

**Conclusions:**

OA increases the risk of CTS, but this is not limited to patients with hand/wrist OA, suggesting a systemic impact of OA on CTS. While the risk appears highest for patients with hand/wrist OA, patients with more distant affected joints like knee or hip also have an increased risk of CTS.

**Supplementary Information:**

The online version contains supplementary material available at 10.1186/s12891-024-07459-1.

## Introduction

Carpal tunnel syndrome (CTS) is an entrapment neuropathy caused by pressure of the median nerve [1, 2], and is characterized by paresthesia, weakness, numbness, and pain occurring in the hand [3]; symptoms can spread to the forearm, upper arm, and shoulders in severe cases [3]. CTS is a progressive condition, and while many patients remain stable (40–62%) or improve spontaneously (23–40%) [4–8], untreated CTS can lead to permanent median nerve damage, and negative impacts on patients' quality of life [9]. Non-surgical interventions, such as change in habits, splinting, ultrasound, and steroid injections may improve symptoms and functional outcomes in some patients [3, 8]; however, over 400,000 carpal tunnel release surgeries are performed each year in the US in patients who cannot be managed with more conservative methods [10]. The outcomes of surgery are highly effective with a success rate of 75–90% [11] and low complication rates (2.6–3.2%). However, revision surgery may be required for persistent, recurring or new symptoms [10]. Furthermore, CTS is associated with a decreased quality of life, loss of productivity, high healthcare costs, and increased societal expenditure for compensation [12, 13].

There are several interacting pathophysiologic mechanisms involved in CTS, including increased pressure in the tunnel, median nerve microcirculation injury, median nerve connective tissue compression, and synovial tissue hypertrophy [9, 14]. Risk factors can be both occupational and medical, including, obesity, genetics, and female sex [14, 15]. Females are hypothesized to be at higher risk due to their smaller relative cross-sectional area of the carpal tunnel compared with men [9]. Other risk factors such as pregnancy, menopause, obesity, renal failure, hypothyroidism, use of oral contraceptives, and congestive heart failure can increase the volume of the synovial sheath within the tunnel. Risk factors including fractures and inherited characteristics can impact the contour of the tunnel, and tumors or lesions can decrease the volume inside the tunnel [14]. Furthermore, neuropathic factors (eg, diabetes and alcoholism) can also play a role in eliciting CTS symptoms through various mechanisms [14]. Several studies have also suggested an association between any arthritis and CTS, or specifically rheumatoid arthritis (RA) and CTS [16, 17], with RA hypothesized to increase the risk of CTS by causing a local inflammatory process of the tendons and tendon sheaths [18].

Osteoarthritis (OA), the most common form of arthritis, affects an estimated 25.6 to 51.87 million US adults [19–21] and has a different underlying pathology from RA. The mechanistic risk for CTS among OA patients therefore likely differs from that of patients with RA. Studies hypothesize that a potential increased risk of CTS related to OA may be due to bony hypertrophy and increased pressure inside the carpal tunnel [17, 22], as well as pathological changes in more distant joints potentially due to inflammatory cytokines released in the circulation rather than locally, as in RA [23].

Few studies have examined a potential association between OA and CTS [17]; these are limited to a study assessing patients with any self-reported OA, and additional studies assessing affected joints in hand, wrist, or spine OA. Conclusions drawn from these studies are limited by the potential for selection bias and confounding, due to their study design (ie, case–control, cross-sectional), small sample sizes, lack of control for any confounding variables, and recency of data (1998–2012). As such, there is a need for additional research to better understand the potential relationship between OA and CTS. While OA in the hand or wrist may predispose to CTS secondary to bony enlargement and/or local inflammation, an association with OA in distant joints may suggest a systemic nature to the disease, at least in some patients.

This study estimated the incidence of CTS in a large US population-based cohort of patients with OA, as well as the risk of CTS among OA overall and by affected joint (hand, knee, hip, wrist, other, and unspecified). In the US, the hand, knee, and hip are the most common joint sites affected by OA [20], and understanding risk in these specific populations is important for early diagnosis and disease management. Furthermore, understanding any differences in CTS risk by joint type will further our understanding of the biological mechanisms which potentially contribute to an association between CTS and OA.

## Methods

### Data source

This population-based cohort study used the Optum Clinformatics Data Mart database, a health claims database encompassing all 50 US states. This database, a de-identified, closed system of administrative health claims, includes enrolment and medical and pharmacy claims of individuals covered by commercial insurance and Medicare Advantage health plans.

### Study population

Patients aged ≥ 45 years and diagnosed with OA between January 1, 2018, and December 31, 2022, were eligible for the OA cohort. OA was defined as having ≥ 1 inpatient or outpatient diagnosis using International Classifications of Diseases, Tenth Revision-Clinical Modification (ICD-10-CM) codes (Additional file 1).

Patients were included in the non-OA cohort (the general population of patients without OA) based on a 25% random sample from the database. The non-OA cohort were individuals who did not have a diagnosis of OA at the index date in 2018–2021 and who had no history of OA during the 12-month pre-index period.

In the OA cohort, the index date was defined as the date of first OA diagnosis where a patient met the eligibility criteria. A random index date was generated for the non-OA cohort to ensure the follow-up times were balanced for both groups. Patients who did not meet the continuous enrolment criteria during the 12-month pre-index period, or who had a history of CTS, were excluded from the study. A history of CTS was defined as receiving at ≥ 1 diagnosis of CTS or undergoing carpal tunnel release surgery in the 12-month pre-index period (Additional file 2).

Patients in both cohorts were followed from the index date to either the date of the first diagnosis of CTS or the first carpal tunnel release procedure, the end of the study period, the end of continuous enrolment, a diagnosis of OA, or death, whichever came first. Patients in the non-OA cohort were censored if they received a diagnosis of OA.

### Outcome and covariates

The outcome of interest was the diagnosis of CTS, which was identified through either one inpatient diagnosis, two outpatient diagnoses, or one procedure encounter of carpal tunnel release surgery, using ICD-10-CM diagnosis and procedure codes (Additional file 2).

Demographic covariates included age, sex, race, and ethnicity (White, Black, Asian, and all Hispanics), payor, and region, and clinical covariates included Charlson Comorbidity Index (CCI), diabetes, hypothyroidism, and RA. The CCI is a tool used to predict mortality based on the presence of a range of comorbidities, with a score of zero signifying the absence of comorbidities. Previous literature has reported that diabetes, hypothyroidism, and RA are risk factors for CTS [15].

The affected OA joint at the index date was determined using ICD-10 codes and was grouped according to site (hand or wrist, knee, hip, shoulder, other, and unspecified). See Additional file 1 for specific definitions.

### Statistical analysis

Descriptive statistics were performed to describe baseline demographic and clinical characteristics in both cohorts. Categorical variables were reported using frequencies and percentages; continuous variables were summarized using means and standard deviations (SDs). To reduce the potential for confounding, baseline characteristics between the two cohorts were balanced using nearest neighbor propensity-score matching (PSM) with a greedy algorithm. Logistic regression was used to estimate propensity score (ie, the likelihood of being diagnosed with OA given observed covariates). The standardized mean difference (SMD) between cohorts was used to demonstrate the balance before and after PSM. A threshold of SMD < 0.25 was used to determine whether covariate balance was achieved after PSM [24, 25].

Incidence rates were defined as the number of new cases of CTS divided by the person-time at risk. These rates were estimated for both the OA and non-OA cohorts before and after PSM. The incidence rates were stratified according to patient characteristics, as well as by the number of affected joints and the type of joint affected by OA within the OA cohort.

Cox proportional hazard regression models were used to estimate the effect of OA on the risk of developing CTS, and hazard ratios (HRs) with 95% confidence intervals (CIs) were reported. In the OA cohort, HRs were stratified by affected joint type on the index date.

Sample selection and creation of analytic variables were performed using the Instant Health Data software (Panalgo, Boston, MA). Statistical analyses were undertaken with R, version 3.2.1 (R Foundation for Statistical Computing, Vienna, Austria) and SAS version 9.4 (SAS Institute Inc, North Carolina, US).

## Results

### Study population

**Fig. 1:**
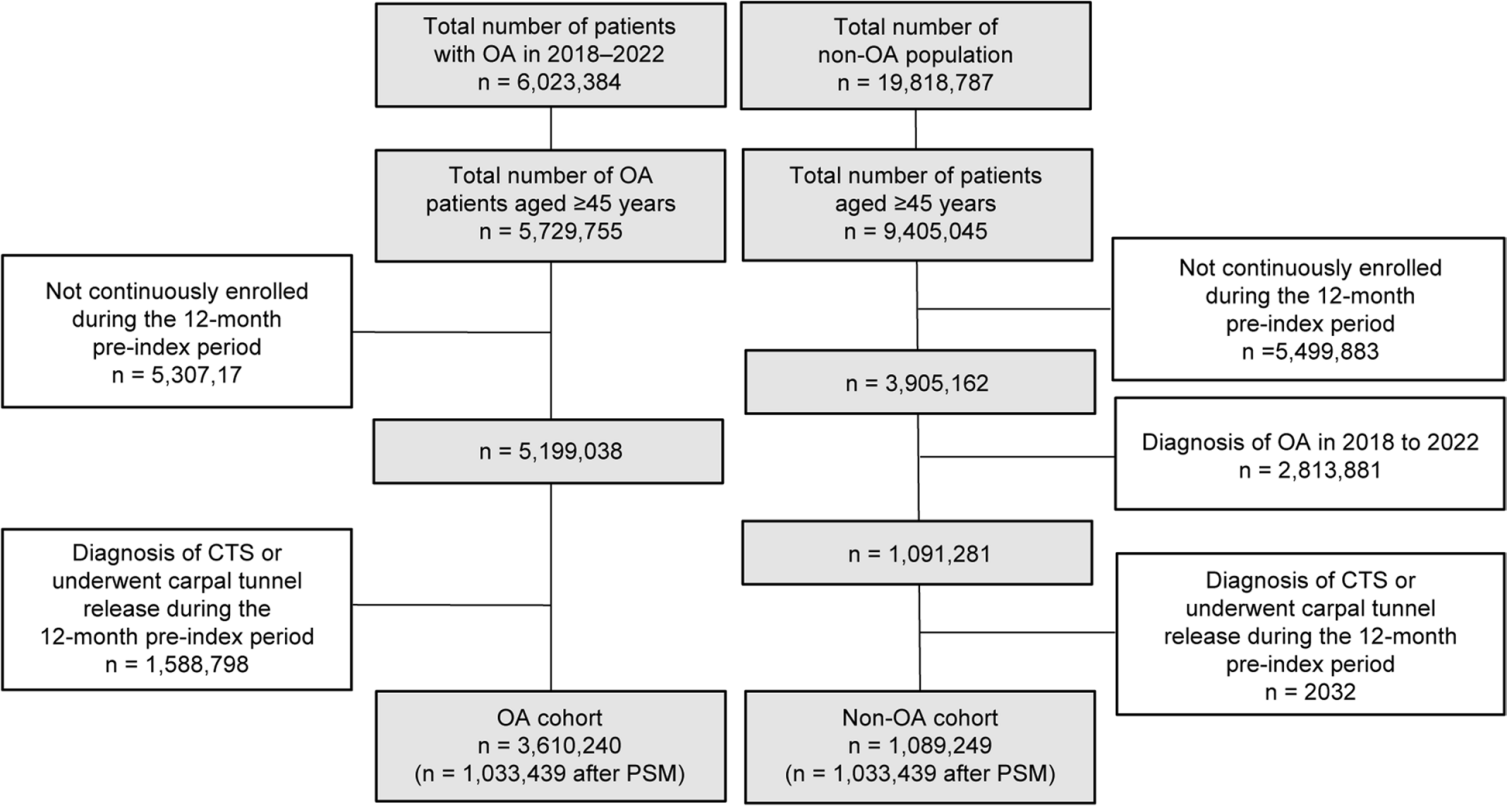
Cohort flow diagram. Patients were included in the OA or non-OA cohort according to the presented criteria. *CTS* Carpal tunnel syndrome, *OA* Osteoarthritis, *PSM *Propensity score matching

A total of 6,023,384 patients with a diagnosis of OA were identified in the Optum database between January 1, 2018, and December 31, 2022. After applying the inclusion and exclusion criteria, 3,610,240 adults remained in the OA cohort. After PSM, 1,033,439 individuals were included in each cohort. The attrition flow for both the OA and non-OA cohorts is depicted in Fig. 1.

Baseline demographics and clinical characteristics varied for OA and non-OA cohorts prior to PSM (Additional file 3). Patients with OA compared with those without OA tended to be older (mean age [SD]: 70.2 [10.5] versus 64.4 [12.0] years), female (60.2% versus 49.8%), and were Medicare patients (75.7% versus 50.7%). Compared with the non-OA cohort, patients with OA also had a higher CCI score (mean CCI [SD]: 1.5 [1.9] versus 0.7 [1.4]) and a higher frequency of comorbidities (hypothyroidism [11.2% versus 4.3%], type 2 diabetes [18.5% versus 7.7%], and RA [4.0% versus 0.8%]). After PSM, all baseline characteristics were balanced between the OA and non-OA cohorts (Table 1).

**Table 1:** Baseline demographics and clinical characteristics of propensity-score matched adults with and without OA^a^

	**Adults with OA (*n*** **= 1,033,439)**	**Adults without OA (*n*** **= 1,033,439)**	**SMD**
Age at index, years, mean (SD)	65.53 (11.11)	64.43 (11.98)	0.09941
Age group, years, *n* (%)			
45–49	90,294 (8.74)	138,910 (13.44)	
50–59	248,524 (24.05)	263,469 (25.49)	
60–69	301,515 (29.18)	250,805 (24.27)	
≥ 70	393,106 (38.04)	380,255 (36.80)	
Sex, *n* (%)			
Female	529,311 (51.22)	516,915 (50.02)	-0.0239
Male	504,152 (48.78)	516,548 (49.98)	
Race, *n* (%)			
Asian	47,265 (4.57)	49,683 (4.81)	-0.0194
Black	105,813 (10.24)	101,664 (9.84)	
White	750,182 (72.59)	745,264 (72.11)	
Hispanic	130,179 (12.60)	136,828 (13.24)	
Payor, *n* (%)			
Commercial	419,590 (40.60)	511,291 (49.47)	0.0979
Medicare	613,849 (59.40)	522,148 (50.53)	
Region, *n* (%)			
Midwest	240,326 (23.28)	213,695 (21.05)	
Northeast	123,090 (11.92)	110,965 (10.93)	
South	449,408 (43.53)	415,851 (40.96)	
West	219,639 (21.27)	274,860 (27.07)	
CCI group at baseline, *n* (%)			0.1257
0–1	796,180 (78.53)	843,124 (83.13)	
1–2	118,944 (11.73)	97,453 (9.61)	
≥ 2	98,780 (9.74)	73,707 (7.27)	
Hypothyroidism, *n* (%)	61,907 (5.99)	45,211 (4.37)	0.0681
Type 2 diabetes mellitus, *n* (%)	105,283 (10.19)	79,826 (7.72)	0.0814
Rheumatoid arthritis, *n* (%)	16,533 (1.60)	8002 (0.77)	0.0658

*CCI* Charlson Comorbidity Index, *OA* Osteoarthritis, *SD* Standard deviation, *SMD* Standardized mean difference

^a^PSM was used to identify matched patients without OA for each patient with OA according to the propensity score. Logistic regression analysis was used to estimate each patient’s propensity score (ie, their likelihood of being diagnosed with OA given observed covariates). Covariates included age, sex, race, CCI, diabetes, hypothyroidism, and rheumatoid arthritis. A caliper of 0.2 on the probability scale was used for matching without replacement

### Incidence of CTS

The incidence rate for CTS for the OA cohort was 7.35 (95% CI 7.21–7.49) per 1000 person-years, higher than the incidence rate for the non-OA cohort 1.44 (95% CI 1.38–1.50) per 1000 person-years (Table 2) after PSM. In both cohorts, females had a slightly higher incidence rate than males. The incidence peaked in the 50–59 years age group, followed by the 45–49, 60–69, and 70 + years age groups. Patients who were Black had the highest incidence rate, followed by those who were White; patients who were Hispanic and Asian had lower incidence rates of CTS.

**Table 2:** Incidence rates of CTS per 1000 person-years among propensity-score matched adults with and without OA^a^

	**OA cohort**			**Non-OA cohort**		
	**CTS cases (*n*)**	**Person-years**	**Incidence rate per 1000 person-years (95% CI)**	**CTS cases (*n*)**	**Person-years**	**Incidence rate per 1000 person-years (95% CI)**
Total	10,341	1,407,101.89	7.35 (7.21–7.49)	2214	1,540,605.02	1.44 (1.38–1.50)
Age group, years						
45–49	807	110,052.20	7.33 (6.84–7.86)	305	216,356.38	1.41 (1.26–1.57)
50–59	2670	317,274.92	8.42 (8.10–8.74)	679	408,599.46	1.66 (1.54–1.79)
60–69	3180	442,719.87	7.18 (6.94–7.44)	541	406,949.92	1.33 (1.22–1.45)
≥ 70	3684	537,054.90	6.86 (6.64–7.08)	689	508,652.86	1.35 (1.26–1.46)
Sex						
Female	5603	748,429.78	7.49 (7.29–7.69)	1199	769,147.64	1.56 (1.47–1.65)
Male	4738	658,672.11	7.19 (6.99–7.40)	1015	771,410.98	1.32 (1.24–1.40)
Race						
Asian	248	61,908.46	4.01 (3.54–4.54)	46	77,790.65	0.64 (0.48–0.85)
Black	1228	151,215.48	8.12 (7.68–8.59)	241	152,418.36	1.72 (1.54–1.93)
White	7885	1,015,503.54	7.76 (7.60–7.94)	1727	1,110,611.95	1.72 (1.65–1.80)
Hispanic	980	178,474.41	5.49 (5.16–5.85)	200	199,737.66	1.08 (0.95–1.24)

*CCI* Charlson Comorbidity Index, *CI* Confidence interval, *CTS* Carpal tunnel syndrome, *OA* Osteoarthritis, *PSM* Propensity score matching

^a^PSM was used to identify matched patients without OA for each patient with OA according to the propensity score. Logistic regression analysis was used to estimate each patient’s propensity score (ie, their likelihood of being diagnosed with OA given observed covariates). Covariates included age, sex, race, CCI, diabetes, hypothyroidism, and rheumatoid arthritis. A caliper of 0.2 on the probability scale was used for matching without replacement

**Fig. 2:**
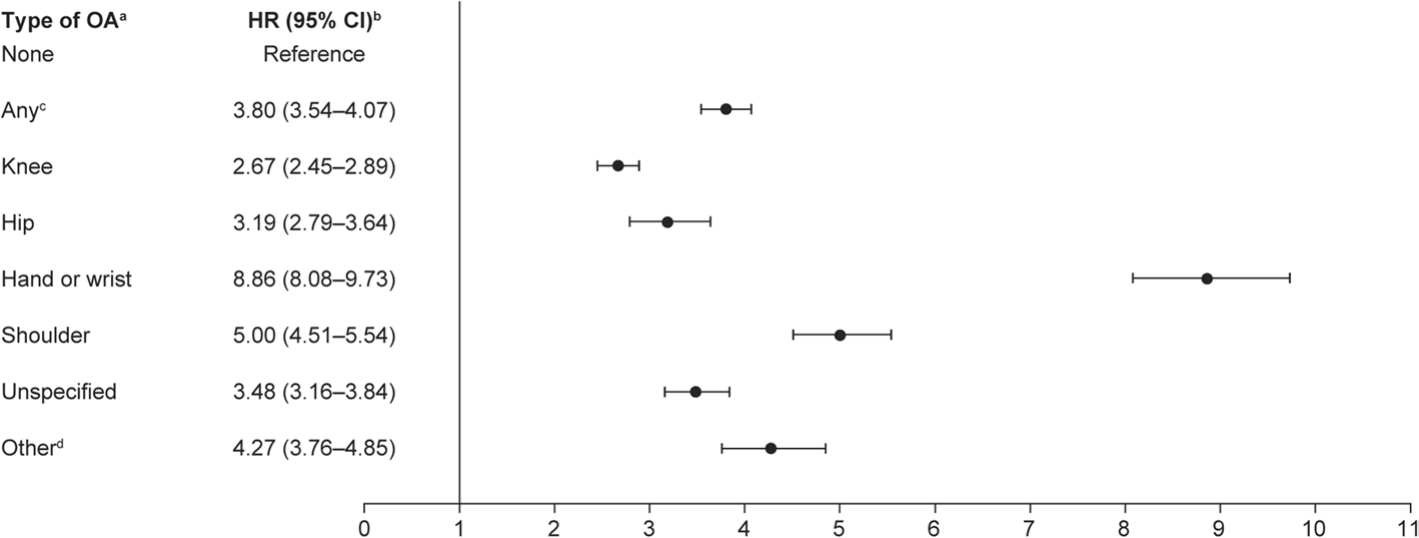
Relative risk of CTS by type of osteoarthritis in propensity score matched cohorts. ^a^Index OA diagnosis, ^b^Propensity-score weighted HR, ^c^For any OA, the types of OA are not mutually exclusive. Patients with ≥ 2 index joints were included in more than one category, ^d^Other OA includes elbow, ankle, and foot. *CI* Confidence interval, *CTS* Carpal tunnel syndrome, *HR* Hazard ratio, *OA* Osteoarthritis

The risk of developing CTS among patients with OA was nearly four times that for patients without OA (HR = 3.80; 95% CI 3.54–4.07) after adjusting for covariates. Hand or wrist OA were associated with the highest risk for CTS compared with the non-OA population (HR = 8.86; 95% CI 8.08–9.73), while knee OA was associated with the lowest risk (HR = 2.67; 95% CI 2.45–2.89) (Table 3 and Fig. 2).

**Table 3:** Incidence rates of CTS per 1000 person-years and risk of CTS by type of osteoarthritis in propensity score matched cohorts

Type of OA^a^	CTS cases (*n*)	Person-years	Incidence rate (95% CI)
None	2214	1,540,605.02	1.44 (1.38–1.50)
Any^b^	10,341	1,407,249.29	7.35 (7.21–7.49)
Knee	3584	620,964.66	5.77 (5.59–5.96)
Hip	691	114,337.30	6.04 (5.61–6.51)
Hand or wrist	2187	130,629.78	16.74 (16.06–17.46)
Shoulder	1327	152,824.91	8.68 (8.23–9.16)
Unspecified	1980	297,043.54	6.67 (6.38–6.97)
Other^c^	763	102,099.93	7.47 (6.96–8.02)

*CI* Confidence interval, *CTS* Carpal tunnel syndrome, *OA* Osteoarthritis

^a^Index OA diagnosis

^b^For any OA, the types of OA are not mutually exclusive. Patients with ≥ 2 index joints were included in more than one category

^c^Other OA includes elbow, ankle, and foot

Patients with multiple affected joints on the index date had a higher incidence rate of CTS compared with those with only one affected joint, although CIs for point estimates overlapped (Additional file 4). Specifically, patients with three or more index joints had an incidence rate of 11.42 (95% CI 7.97–16.40) and those with two index joints had an incidence rate of 8.74 (95% CI 7.88–9.70); these were both higher than the incidence rate of 7.32 (95% CI 7.18–7.47) for patients with only one affected index joint. This increased incidence with an increasing number of affected joints remained consistent even when patients with hand or wrist index joints were excluded (one index joint: 6.44 [95% CI 6.30–6.58]; two index joints: 7.22 [95% CI 6.31–8.25]; ≥ 3 index joints, 9.85 [95% CI 5.55–17.63]).

## Discussion

Our population-based cohort study found a substantial increased risk of developing CTS among patients with OA compared with those without OA, with an increased risk noted across all OA joint types. Similarly, a prospective longitudinal cohort study in Wisconsin found that approximately twice as many patients with self-reported OA were diagnosed with CTS compared with patients without OA (HR = 2.2; 95% CI 0.92–5.33) in a manufacturing setting [26]. Additional studies have focused on specific joints but are limited to assessing risk among patients with affected wrist, spine, and unspecified joints. Several studies have shown that wrist OA increases the risk of CTS: one UK case control study reported an odds ratio (OR) of 1.89 (95% CI 1.65–2.17) [15]; and another reported ORs varying across locations in the wrist from 5.12 to 44.10 [22]. Similarly, spine OA (OR = 2.88; 95% CI 1.45–5.74) and unspecified OA (OR = 1.90; 95% CI 1.00–3.61) have also been reported to elevate CTS risk [22]. Researchers hypothesize that the observed higher risk of CTS in wrist OA can be attributed to degenerative narrowing of carpal tunnel space in the wrist, triggering CTS symptoms [17, 22].

Given the observed associations with joints affected by OA other than the hand or wrist, our study suggests there may be a systemic impact of OA on the risk of CTS. This potential systemic impact is further supported by our exploratory analysis suggesting a higher incidence rate of CTS in patients with multiple affected joints, even when the hand or wrist is not involved. It is not clear whether these associations are the result of increased circulating inflammatory factors (such as IL-6) [27] or a predisposition to multi-site OA secondary to metabolic or genetic factors (like GDF-5) [28, 29]; further study would be required to address this question.

Estimates of CTS incidence in the US population have primarily been limited to specific high-risk occupational cohorts, such as those in military, manufacturing, industrial, and clerical work. These estimates range from 3.98 to 25.5 cases per 1000 person-years [30–32]. Given our population-based cohort study approach, we were also able to estimate the incidence rate of CTS in the US general population, which is of interest to understand the absolute magnitude of risk in this population. The incidence of CTS in our population of US adults ≥ 45 years of age with no diagnosis of OA (1.44 per 1000 person-years overall; 1.56 and 1.32 per 1000 person-years for females and males, respectively) was similar to estimates seen in the general populations of other developed countries including Sweden (1.99–3.24 per 1000 person-years for females and 0.85–1.87 per 1000 person–years for males) [33] and Korea (1.38 per 1000 person-years [95% CI 1.35–1.42]) [34].

Studies have identified several demographic and clinical characteristics, as well as comorbidities, that increase the risk of CTS. By using PSM we were able to adjust for these risks, thereby enabling us to better isolate the OA-associated risk of developing CTS. While we included patients with RA in the analysis to capture a broader population, we adjusted for it using PSM. To assess for residual confounding, we performed a sensitivity analysis excluding patients with RA. Results remained similar, indicating that our findings were robust (Additional file 5). In addition, patients with OA may be likely to visit a physician due to their OA diagnosis or the fact that patients with OA tend to have more comorbidities than the general population [35]. This may increase the likelihood of CTS diagnosis among these patients. We therefore included baseline CCI score in our propensity score matching in an attempt to alleviate the difference in healthcare utilization between the OA and non-OA populations, and mitigate its impact on the diagnosis of CTS in these two cohorts.

Our study was based on a large sample drawn from a geographically representative US population, using claims to capture CTS. While this is a more rigorous approach than identifying the condition based on patient self-reporting, diagnostic criteria for CTS may vary by physician specialty and experience level [3]. Given the intermittent nature of CTS symptoms, which often peak with years of gap in between, CTS cases may not immediately be identified resulting in an over or underestimate of CTS and any given time. Furthermore, studies using administrative claims data have several inherent limitations, which are applicable to our study. Firstly, administrative claims data do not record the rationale or the criteria contributing to the diagnosis of CTS; results of physical findings, diagnostic questionnaires, imaging, or electrophysiological data are not available in claims databases. Secondly, the presence of ICD-10-CM codes was used to identify diagnoses of OA and CTS. However, the specificity and sensitivity of ICD-10 codes for these two diseases have not been validated in the literature. Patients with CTS and OA exhibit similar symptoms, which may result in both OA and CTS codes being assigned to these patients. A more restrictive algorithm (ie, one inpatient or two outpatients or one procedure of carpal tunnel release) was used to identify CTS to increase specificity [36, 37]. Lastly, it should be noted that the commercial data only included patients covered by private insurance and Medicare and does not represent a random sample of US patients. Thus, the findings from this study may not be generalizable to the entire US population [38].

In addition, there may be some misclassification of the number of affected joints. While we were able to stratify incidence rate by the types and number of affected joints, and to assess risk by specific affected joints, these were categorized according to first affected joint and any joints diagnosed during follow-up were not captured in this categorization. Diagnosing hand OA based on conventional radiography (ie, X-rays) can be challenging, especially in the early stages [39], due to its limitations in imaging soft tissues and subchondral structures as well as in detecting erosions [39–42]. This makes it difficult to detect all affected joints, particularly those in the hand or wrist, leading to the possibility that some cases of hand or wrist OA may be underestimated in this database. Approximately 25% of adults had unspecified types of OA, which restricted our capability to link a specific type of OA to the incidence of CTS. Lastly, the Optum database does not record certain risk factors such as body mass index, occupations involving high force and repetitive movements, or personal behaviors like alcohol consumption, which may confound the relationship between OA and CTS. Despite the lack of data on occupational factors, our results demonstrate an increased incidence of CTS in the population aged ≥ 70 years. This finding suggests that current occupation does not entirely account for the association between OA and CTS, especially considering that individuals in this age group are less likely to be part of the active working population. However, the Optum database does not provide information on individuals’ past occupational history. It remains possible that a long-standing history of high-risk occupations could potentially contribute to the onset of CTS. Therefore, while our findings indicate a potential link between OA and CTS that extends beyond current occupational factors, the lack of data on previous work history remains a limitation of our analysis.

## Conclusions

This is the first population-based cohort study in the US to provide incidence rates of CTS in adults with OA, and to examine the risk of OA by affected joints. Our study not only reports that OA increases the risk of CTS, but also suggests that this risk is not limited to patients with hand or wrist OA, suggesting a systemic impact of OA on CTS. While risk appears to be highest for patients with affected hand or wrist joints, those with more distant affected joints like the knee or hip also have an increased risk. Results are also suggestive of an increased risk among patients with an increasing number of affected joints.

### Supplementary Information

**Supplementary Material 1.**

**Supplementary Material 2.**

**Supplementary Material 3.**

**Supplementary Material 4.**

**Supplementary Material 5.**

## Data Availability

Qualified researchers may request access to study documents (including the literature review, and statistical analysis plan) that support the methods and findings reported in this manuscript. Individual anonymized participant data will be considered for sharing, if there is legal authority to share the data and there is not a reasonable likelihood of participant re-identification. Requests should be submitted to https://vivli.org/.

## Abbreviations

CCI: Charlson Comorbidity Index
CI: Confidence interval
CTS: Carpal tunnel syndrome
HR: Hazard ratio
ICD-10-CM: International Classification of Diseases, Tenth Revision, Clinical Modification
OA: Osteoarthritis
OR: Odds ratio
PSM: Propensity score matching
RA: Rheumatoid arthritis
SD: Standard deviation
SMD: Standardized mean difference
