# Supplementary material for: Risk of carpal tunnel syndrome among patients with osteoarthritis: a US population-based study
Source: BMC Musculoskelet Disord. 2024 Jun 15;25:468. doi: 10.1186/s12891-024-07459-1 (PMC11179394; doi:10.1186/s12891-024-07459-1)
Supplement: Supplementary file 1 — Supplementary Material 1. [file 12891_2024_7459_MOESM1_ESM.docx]

Additional file 1. ICD-10-CM codes for OA

| **OA** | **ICD-10-CM codes** |
| --- | --- |
| OA (any site) | M15-M19 OA  M15  Polyosteoarthritis  M16  OA of hip  M17  OA of knee  M18  OA of first carpometacarpal joint  M19  Other and unspecified OA |
| Knee OA | M15.0 Primary generalized (osteo)arthritis  M15.3 Secondary multiple arthritis  M15.4 Erosive (osteo)arthritis  M15.8 Other polyosteoarthritis  M15.9 Polyosteoarthritis, unspecified  M170 Bilateral primary OA of knee  M17.1, M17.10, M17.11, M17.12 Unilateral primary OA of knee  M17.2 Bilateral post-traumatic OA of knee  M17.30, M17.31, M17.32 Unilateral post-traumatic OA of knee  M17.4 Other bilateral secondary OA of knee  M17.5 Other unilateral secondary OA of knee  M17.9 Primary OA of other joints |
| Hip OA | M1610 Unilateral primary OA, unspecified hip  M1611 Unilateral primary OA, right hip  M1612 Unilateral primary OA, left hip  M1630 Unilateral OA resulting from hip dysplasia, unspecified hip  M1631 Unilateral OA resulting from hip dysplasia, right hip  M1632 Unilateral osteoarthritis resulting from hip dysplasia, left hip  M164 Bilateral post-traumatic OA of hip  M1650 Unilateral post-traumatic OA, unspecified hip  M1651 Unilateral post-traumatic OA, right hip  M1652 Unilateral post-traumatic OA, left hip  M166 Other bilateral secondary OA of hip  M167 Other unilateral secondary OA of hip  M169 OA of hip, unspecified |
| Hand or Wrist OA | M15.1 Heberden's nodes (with arthropathy)  M15.2 Bouchard's nodes (with arthropathy)  M18.0 Bilateral primary OA of first carpometacarpal joints  M18.1 M18.1 Unilateral primary OA of first carpometacarpal joint [non-specific code]  M18.10 Unilateral primary OA of first carpometacarpal joint, unspecified hand  M18.11 Unilateral primary OA of first carpometacarpal joint, right hand  M18.12 Unilateral primary OA of first carpometacarpal joint, left hand  M18.2 Bilateral post-traumatic OA of first carpometacarpal joints  M18.3 Unilateral post-traumatic OA of first carpometacarpal joint [non-specific code]  M18.30 Unilateral post-traumatic OA of first carpometacarpal joint, unspecified hand  M18.31 Unilateral post-traumatic OA of first carpometacarpal joint, right hand  M18.32 Unilateral post-traumatic OA of first carpometacarpal joint, left hand  M18.4 Other bilateral secondary OA of first carpometacarpal joints  M18.5 Other unilateral secondary OA of first carpometacarpal joint [non-specific code]  M18.50 Other unilateral secondary OA of first carpometacarpal joint, unspecified hand  M18.51 Other unilateral secondary OA of first carpometacarpal joint, right hand  M18.52 Other unilateral secondary OA of first carpometacarpal joint, left hand  M18.9 OA of first carpometacarpal joint, unspecified  M19.03 Primary OA, wrist [non-specific code]  M19.031 Primary OA, right wrist  M19.032 Primary OA, left wrist  M19.039 Primary OA, unspecified wrist  M19.04 Primary OA, hand [non-specific code]  M19.041 Primary OA, right hand  M19.042 Primary OA, left hand  M19.049 Primary OA, unspecified hand  M19.13 Post-traumatic OA, wrist [non-specific code]  M19.131 Post-traumatic OA, right wrist  M19.132 Post-traumatic OA, left wrist  M19.139 Post-traumatic OA, unspecified wrist  M19.14 Post-traumatic OA, hand [non-specific code]  M19.141 Post-traumatic OA, right hand  M19.142 Post-traumatic OA, left hand  M19.149 Post-traumatic OA, unspecified hand  M19.23 Secondary OA, wrist [non-specific code]  M19.231 Secondary OA, right wrist  M19.232 Secondary OA, left wrist  M19.239 Secondary OA, unspecified wrist  M19.24 Secondary OA, hand [non-specific code]  M19.241 Secondary OA, right hand  M19.242 Secondary OA, left hand  M19.249 Secondary OA, unspecified hand |
| Shoulder OA | M19.01 Primary OA, shoulder [non-specific code]  M19.011 Primary OA, right shoulder  M19.012 Primary OA, left shoulder  M19.019 Primary OA, unspecified shoulder  M19.11 Post-traumatic OA, shoulder [non-specific code]  M19.111 Post-traumatic OA, right shoulder  M19.112 Post-traumatic OA, left shoulder  M19.119 Post-traumatic OA, unspecified shoulder  M19.21 Secondary OA, shoulder [non-specific code]  M19.211 Secondary OA, right shoulder  M19.212 Secondary OA, left shoulder  M19.219 Secondary OA, unspecified shoulder |
| Unspecified OA | M19.9 OA, unspecified site  M19.90 Unspecified OA, unspecified site  M19.91 Primary OA, unspecified site  M19.92 Post-traumatic OA, unspecified site  M19.93 Secondary OA, unspecified site |
| Other OA (elbow, ankle/foot) | M19.02 Primary OA, elbow [non-specific code]  M19.021 Primary OA, right elbow  M19.022 Primary OA, left elbow  M19.029 Primary OA, unspecified elbow  M19.07 Primary OA ankle and foot [non-specific code]  M19.071 Primary OA, right ankle and foot  M19.072 Primary OA, left ankle and foot  M19.079 Primary OA, unspecified ankle and foot  M19.09 Primary OA, other specified site  M19.1 Post-traumatic OA of other joints [non-specific code]  M19.12 Post-traumatic OA, elbow [non-specific code]  M19.121 Post-traumatic OA, right elbow  M19.122 Post-traumatic OA, left elbow  M19.129 Post-traumatic OA, unspecified elbow  M19.17 Post-traumatic OA, ankle and foot [non-specific code]  M19.171 Post-traumatic OA, right ankle and foot  M19.172 Post-traumatic OA, left ankle and foot  M19.179 Post-traumatic OA, unspecified ankle and foot  M19.19 Post-traumatic OA, other specified site  M19.22 Secondary OA, elbow [non-specific code]  M19.221 Secondary OA, right elbow  M19.222 Secondary OA, left elbow  M19.229 Secondary OA, unspecified elbow  M19.27 Secondary OA, ankle and foot [non-specific code]  M19.271 Secondary OA, right ankle and foot  M19.272 Secondary OA, left ankle and foot  M19.279 Secondary OA, unspecified ankle and foot  M19.29 Secondary OA, other specified site |

Codes for the following comorbidities are available on request: hypothyroidism; rheumatoid arthritis; and type 2 diabetes mellitus.

ICD-10-CM, International Classification of Diseases, Tenth Revision, Clinical Modification; OA, osteoarthritis.
