# Supplementary material for: Risk of carpal tunnel syndrome among patients with osteoarthritis: a US population-based study
Source: BMC Musculoskelet Disord. 2024 Jun 15;25:468. doi: 10.1186/s12891-024-07459-1 (PMC11179394; doi:10.1186/s12891-024-07459-1)
Supplement: Supplementary file 2 — Supplementary Material 2. [file 12891_2024_7459_MOESM2_ESM.docx]

Additional file 2. ICD-10-CM codes for CTS and CPT codes/ICD-10-CM codes for carpal tunnel release

| **Procedure** | **Category** | **Codes** |
| --- | --- | --- |
| CTS | ICD-10-CM | G56.0 CTS  G56.00 CTS, unspecified upper limb  G56.01 CTS, right upper limb  G56.02 CTS, left upper limb  G56.03 CTS, bilateral upper limbs |
| Carpal tunnel release | CPT | 29848 Endoscopy, wrist, surgical, with release of transverse carpal ligament  64721 Neuroplasty and/or transposition; median nerve at carpal tunnel |
|  | ICD-10-CM | 01N50ZZ Release median nerve, open approach  01N53ZZ Release median nerve, percutaneous approach  01N54ZZ Release median nerve, percutaneous endoscopic approach  0PNM0ZZ Release right carpal, open approach  0PNM3ZZ Release right carpal, percutaneous approach  0PNM4ZZ Release right carpal, percutaneous endoscopic approach  0PNN0ZZ Release left carpal, open approach  0PNN3ZZ Release left carpal, percutaneous approach  0PNN4ZZ Release left carpal, percutaneous endoscopic approach |

CPT, Current Procedural Terminology; CTS, carpal tunnel syndrome; ICD-10-CM, International Classification of Diseases, Tenth Revision, Clinical Modification.
