# Supplementary material for: Risk of carpal tunnel syndrome among patients with osteoarthritis: a US population-based study
Source: BMC Musculoskelet Disord. 2024 Jun 15;25:468. doi: 10.1186/s12891-024-07459-1 (PMC11179394; doi:10.1186/s12891-024-07459-1)
Supplement: Supplementary file 3 — Supplementary Material 3. [file 12891_2024_7459_MOESM3_ESM.docx]

Additional file 3. Baseline demographics and clinical characteristics for the unmatched cohorts

|  |  | **OA cohort**  **(n = 3,610,240)** | **Non-OA cohort**  **(n = 1,089,249)** | **Standardized mean difference^a^** |
| --- | --- | --- | --- | --- |
| Age at index, years | |  |  |  |
|  | Mean (SD) | 70.2 (10.5) | 64.4 (12.0) | 0.5531 |
|  | Median (IQR) | 71.0 (63.0–78.0) | 65.0 (54.0–73.0) |  |
| Age group, n (%) | |  |  |  |
|  | 45–49 | 131,462 (3.64) | 148,800 (13.66) |  |
|  | 50–59 | 502,811 (13.93) | 275,769 (25.32) |  |
|  | 60–69 | 988,667 (27.39) | 263,590 (24.20) | NA |
|  | 70+ | 1,987,300 (55.1) | 401,090 (36.8) |  |
| Gender, n (%) | |  |  |  |
|  | Female | 2,171,878 (60.16) | 542,197 (49.78) | 0.2179 |
|  | Male | 1,438,089 (39.84) | 546,960 (50.22) |  |
| Race, n (%) | |  |  |  |
|  | Asian | 91,684 (2.66) | 54,598 (5.26) | 0.0661 |
|  | Black | 416,956 (12.08) | 101,672 (9.79) |  |
|  | Caucasian | 2,576,768 (74.65) | 745,318 (71.77) |  |
|  | Hispanic | 366,462 (10.62) | 136,831 (13.18) |  |
| Payor, n (%) | |  |  | NA |
|  | Commercial | 878,002 (24.32) | 536,868 (49.29) |  |
|  | Medicare | 2,732,238 (75.68) | 552,381 (50.71) |  |
| Region, n (%) | |  |  |  |
|  | Midwest | 819,194 (22.71) | 222,039 (20.79) | NA |
|  | Northeast | 451,889 (12.53) | 116,404 (10.90) |  |
|  | South | 1,576,126 (43.69) | 434,514 (40.69) |  |
|  | West | 760,355 (21.08) | 294,880 (27.61) |  |
| CCI group at baseline, n (%) | |  |  |  |
|  | <1 | 2,272,136 (65.99) | 890,213 (83.27) | 0.3998 |
|  | ≥1, <2 | 616,620 (17.91) | 77,185 (7.22) |  |
|  | ≥2 | 554,260 (16.10) | 101,681 (9.51) |  |
| Comorbidity, n (%) | |  |  |  |
|  | Type 2 diabetes mellitus | 668,477 (18.52) | 83,395 (7.66 | 0.2787 |
|  | Hypothyroidism | 404,775 (11.21) | 47,137 (4.33) | 0.2179 |
|  | Rheumatoid arthritis | 143,552 (3.98) | 8,315 (0.76%) | 0.1644 |
| Number of affect joints on index date, n (%)^b^ | |  |  |  |
|  | 1 | 3,384,736 (96.71) |  |  |
|  | 2 | 107,273 (3.07) |  |  |
|  | 3+ | 7,782 (0.22) |  |  |
| Index knee OA, n (%) | | 1,428,071 (39.56) |  |  |
| Index hand or wrist OA, n (%) | | 351,902 (9.75) |  |  |
| Index hip OA, n (%) | | 309,893 (8.58) |  |  |
| Index shoulder OA, n (%) | | 395,659 (10.96) |  |  |
| Index other OA, n (%) | | 278,348 (7.71) |  |  |
| Index unspecified OA, n (%) | | 859,480 (23.81) |  |  |

^a^Standardized mean differences are reported for only covariates that were included in propensity score matching, but before matching. Covariates included age, gender, race, CCI, diabetes, hypothyroidism, rheumatoid arthritis.

^b^Date of first OA diagnosis during the study period.

CCI, Charlson Comorbidity Index; IQR, interquartile range; OA, osteoarthritis; SD, standard deviation.
