# Supplementary material for: Risk of carpal tunnel syndrome among patients with osteoarthritis: a US population-based study
Source: BMC Musculoskelet Disord. 2024 Jun 15;25:468. doi: 10.1186/s12891-024-07459-1 (PMC11179394; doi:10.1186/s12891-024-07459-1)
Supplement: Supplementary file 4 — Supplementary Material 4. [file 12891_2024_7459_MOESM4_ESM.docx]

Additional file 4. Incidence rates of CTS per 1000 person-years in patients with multiple affected joints in OA population

|  | **Incidence rate per 1000 person-years (95% CI)** |
| --- | --- |
| Number of affected index joints |  |
| 1 | 7.32 (7.18–7.47) |
| 2 | 8.74 (7.88–9.70) |
| ≥3 | 11.42 (7.97–16.40) |
| Number of affected index joints without hand or wrist |  |
| 1 | 6.44 (6.30–6.58) |
| 2 | 7.22 (6.31–8.25) |
| ≥3 | 9.85 (5.55–17.63) |

These results were based on the OA population, before propensity score matching.

CI, confidence interval; CTS, carpal tunnel syndrome; OA, osteoarthritis.
