# Supplementary material for: Risk of carpal tunnel syndrome among patients with osteoarthritis: a US population-based study
Source: BMC Musculoskelet Disord. 2024 Jun 15;25:468. doi: 10.1186/s12891-024-07459-1 (PMC11179394; doi:10.1186/s12891-024-07459-1)
Supplement: Supplementary file 5 — Supplementary Material 5. [file 12891_2024_7459_MOESM5_ESM.docx]

Additional file 5. Relative risks of CTS among patients with OA by type of OA: comparison of hazard ratios between primary model and sensitivity analysis excluding RA

|  | **Hazard ratios (95% CI)^b^** | |
| --- | --- | --- |
| **Type of OA^a^** | **Primary model** | **Sensitivity analysis^d^** |
| None | Reference | Reference |
| Any | 3.80 (3.54–4.07) | 3.80 (3.55–4.07) |
| Knee | 2.67 (2.45–2.89) | 2.67 (2.46–2.89) |
| Hip | 3.19 (2.79–3.64) | 3.18 (2.78–3.64) |
| Hand or wrist | 8.86 (8.08–9.73) | 8.90 (8.11–9.77) |
| Shoulder | 5.00 (4.51–5.54) | 5.01 (4.52–5.56) |
| Unspecified | 3.48 (3.16–3.84) | 3.47 (3.15–3.83) |
| Other^c^ | 4.27 (3.76–4.85) | 4.26 (3.75 –4.84) |

^a^Index OA diagnosis.

^b^Propensity-score weighted hazard ratiog.

^c^Other OA includes elbow, ankle, and foot.

^d^Sensitivity analysis excluded patients with RA from the analysis.

CI, confidence interval; CTS, carpal tunnel syndrome; OA, osteoarthritis; RA, rheumatoid arthritis.
